# *Acinetobacter baumannii* Infection and IL-17 Mediated Immunity

**DOI:** 10.1155/2016/9834020

**Published:** 2016-02-09

**Authors:** Zihe Yan, Junjun Yang, Renjing Hu, Xichi Hu, Kong Chen

**Affiliations:** ^1^Department of Clinical Laboratory, The Affiliated Wuxi Second Hospital, Nanjing Medical University, Wuxi, Jiangsu 214002, China; ^2^Richard King Mellon Foundation Institute for Pediatric Research, Children's Hospital of Pittsburgh of UPMC, Pittsburgh, PA 15224, USA

## Abstract

*Acinetobacter baumannii* is a significant cause of severe hospital-acquired infections with a recent rise in multidrug-resistant infections involving traumatic wounds of military personnel. The interleukin-17 (IL-17) pathway is essential for neutrophil recruitment in response to a variety of pathogens, while the control of* A. baumannii* infection is known to be dependent on neutrophils. This suggests that IL-17 may play an important role in* A. baumannii* infection; however, this has yet to be studied. Here, we summarize the recent advances in understanding the host-pathogen interaction of* A. baumannii* and propose a potential role of the IL-17 pathway in generating a protective immune response.

## 1. Introduction of* A. baumannii*

*Acinetobacter baumannii* is microbiologically characterized as a rod-shaped, aerobic, pleomorphic, and nonmotile Gram-negative bacterium.* A. baumannii* can survive in natural environments such as soil and water for a prolonged period and mainly infect immunocompromised patients. This opportunistic organism is also known to be resistant to disinfectants and persist in hospital and health care facilities. As a consequence,* A. baumannii* has emerged as a major pathogen causing both community-acquired and nosocomial infections [1].

*A. baumannii* presents as an infection of the skin and soft tissue and causes pneumonia [2]. This pathogen has been strongly associated with wound infections of soldiers serving in Iraq and Afghanistan [3].* A. baumannii* isolates were recovered from various tissues including respiratory tract, blood, flesh wounds, and urinary tract [4]. Many infections were caused by multidrug- and pandrug-resistant strains; this calls for the urgent need of new preventive and therapeutic options against this emerging threat.

Owing to drug resistance to all commonly used Gram-negative antibiotics,* A. baumannii* has generated an increase in research interest [5, 6]. Genomic analyses of multidrug-resistant isolates suggest that these drug resistance genes could be acquired from other Gram-negative species [7]. These studies have revealed that the major drug-resistant mechanisms are through several genes including *β*-lactamases, carbapenemases, DNA gyrase, dihydropteroate synthase/reductase, and efflux pumps [7]. This list is expected to become longer with the current abuse of antibiotics in clinical settings. Thus, the concept of therapeutic approaches that target host responses including the immune response has become more appealing.

## 2. Host Innate Immune Responses against* A. baumannii*

Although our understanding of the epidemiology, mechanisms of antibiotic resistance, and persistence of* A. baumannii* in various environments has advanced, the pathological characteristics are much less studied. Specifically, the immune pathways that are critical to host defense against* A. baumannii* are far from being well understood.

Since* A. baumannii* is a Gram-negative bacterium, it is not surprising that lipopolysaccharide (LPS), a highly immunostimulatory molecule on its surface, induces strong responses from mouse splenocytes and engineered human cells including the human monocytic cell line, THP-1 cells [8, 9]. LPS is recognized by TLR4 and activates NF-*κ*B, which leads to the secretion of MIP-2 and KC/IL-8 and subsequent neutrophil recruitment [9, 10]. TLR2 also seemed to contribute to the inflammatory response to* A. baumannii*, but the results are conflicting: in one report, TLR2-deficient mice exhibit accelerated neutrophil influx to the lungs, improved elimination of* A. baumannii*, and reduced proinflammatory responses. This suggests that TLR2 signaling may shift the immune response from protective to pathological [10]. Other studies have shown that purified TLR2 ligands from* A. baumannii* are immunostimulatory and result in the activation of NF-*κ*B and secretion of IL-8 [9, 11]. Variability in mouse versus human models could potentially explain the conflicting observations but could also be explained by contamination in the TLR ligands during purification.

Depletion of complement, macrophages, or neutrophils separately increased bacterial burden in an infection model with a hypovirulent strain but depletion of these compartments of immune system was insufficient to change lethality [12]. Although the mechanism is not clear, the loss of Fus1, a tumor suppressor protein, in mice significantly increased resistance to* A. baumannii* pneumonia [13]. However, depletion of neutrophils eliminates the enhanced antibacterial clearance of the* Fus1* ^−/−^ mice, further underscoring the importance of neutrophils in the host response to* A. baumannii* pneumonia. These data collectively suggest that neutrophils are an important cellular compartment that is involved in the controlling of* A. baumannii* infection. Based on the compelling evidence on the regulation of neutrophil recruitment by the IL-17 pathway, we hypothesize that the IL-17 producing T helper cells (Th17) play a role in mediating* A. baumannii* clearance.

## 3. IL-17 Pathway in Host Defense at Barrier Tissues

Effector CD4+ T cells differ in their phenotypes depending on differentiating conditions and can be categorized into various lineages [14]. Th1 cells make IFN-*γ* as their signature cytokine, are potent IL-2 producers, and frequently coexpress TNF-*α*. By contrast, Th2 cells do not produce IFN-*γ* but are specialized in making cytokines IL-4, IL-5, and IL-13. The Th1/Th2 paradigm was a dominating theory in the field of T-cell immunology for more than 15 years until 2003, when a series of publications demonstrated a third distinctive effector lineage of CD4+ T cells, Th17 cells, discovered in mouse models of autoimmune encephalitis [15–20]. Most Th17 cells were found to reside in barrier tissues, including respiratory and intestinal tracts as well as the skin. Signature cytokines of Th17 cells include IL-17A, IL-17F, IL-22, and IL-26 (specific for humans) and these canonical cytokines produced by the classical Th17 (CD4+ IL-17 producing cells) and non-Th17 cells including *γδ*-T cells and innate lymphoid cells play critical roles in regulating tissue homeostasis and inflammation as well as antimicrobial responses upon infections caused by pathogens. Detailed roles and functions of IL-17 in host defense at three different mucosal sites including lung, digestive tract, and skin will be discussed below.

### 3.1. Lung

The pivotal roles of IL-17/IL-17 receptor signaling in the context of host defense against bacterial and fungal pathogens are very well recognized and appreciated even before the discovery of the Th17 lineage [21]. In pulmonary infection models, mice deficient in either IL-17 or IL-17RA have increased susceptibility to Gram-negative bacteria, such as* Klebsiella pneumoniae* [21] and* Mycoplasma pneumonia *[22]. During primary infection, IL-17 signals through the heterodimeric receptor IL-17RA/IL-17RC and promotes the production of CXC chemokines such as CXCL1, CXCL2, and CXCL5. These chemokines are critical for recruiting neutrophils which can ultimately clear the bacteria. IL-17 is also critical for the optimal production of G-CSF, an important cytokine that not only prolongs the survival of neutrophils but also improves the function of neutrophils. Th17 cells are also known for their ability to mediate serotype-independent protection in mouse models of* K. pneumoniae* [23],* Streptococcus pneumoniae* [24], and* Pseudomonas aeruginosa* [25]. In these models, Th17 cells have been shown to recognize antigens that are conserved among different bacterial species and provide broader protection upon secondary infection. It has been hypothesized that antigen-specific memory Th17 cells confer a host advantage by providing heterologous mucosal immunity through recognition of conserved antigens among different species of pathogens [23].

### 3.2. Digestive Tract

Th17 cytokines also play critical roles in the digestive system. The expression of IL-17 and IL-22 increases at other mucosal sites after infection with a number of pathogens including intestinal infections with* Citrobacter rodentium* [26–28] or* Salmonella* Typhimurium [29, 30]. The primary roles of IL-17 and/or IL-22 in these models are to control the infection within the mucosa and prevent the dissemination of these pathogens. In the* Citrobacter rodentium* infection model, which mimics infections by attaching and effacing (A/E) bacterial pathogens in humans, IL-22 is required for the colonic epithelial production of antimicrobial proteins, including RegIIIbeta and RegIIIgamma. The IL-22 dependent antimicrobial proteins are crucial in reducing intestinal epithelial damage and decreasing bacterial burden. Th17 cells and IL-17 receptor signaling are also essential for host defense against oral candidiasis caused by* Candida albicans* [31]. Upon oral* Candida* infection, Th17 signature genes including CXC chemokines and beta defensin-3 are strongly induced while IL-17RA deficient mice have more severe oropharyngeal candidiasis as compared to wild type mice.

### 3.3. Skin

In humans, skin is another important anatomical barrier from pathogens and* Staphylococcus aureus* is the most common cause of infection at skin. IL-17 has been shown to be critical in recruiting neutrophils in a skin infection model although the primary cellular source appeared to be epidermal *γδ*-T cells [32]. In this mouse model of* S. aureus* skin infection, neutrophil recruitment to the infection sites was dependent on epidermal *γδ*-T-cell production of IL-17 and this IL-17 induction is controlled by signals from IL-1, TLR2, and IL-23 as IL-17 production upon* S. aureus* infection is diminished in the* Il1r1* ^−/−^,* Tlr2* ^−/−^, and* Il23a* ^−/−^ mice but not* Il12a* ^−/−^ mice.

## 4. Linking Th17 Responses to* A. baumannii*

**Figure 1:**
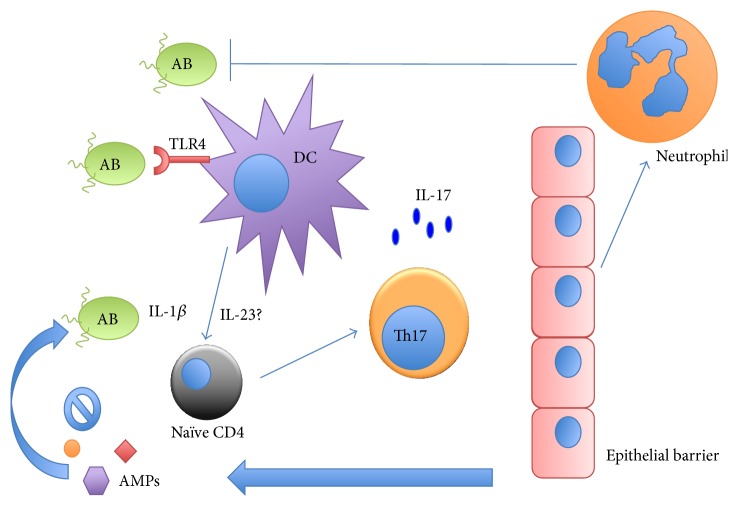
Potential role of Th17 cells in* A. baumannii* (AB) infection. As depicted above, TLR ligands of AB can activate TLRs on DCs and/or macrophages from the host and provoke the release of proinflammatory cytokines such as IL-1*β* and possibly IL-23. Naïve CD4 T cells differentiate into effector Th17 cells and produce Th17 signature cytokines. These cytokines include IL-17 signal epithelial cells at barrier tissues and exert antimicrobial function through 2 possible mechanisms: recruiting neutrophils and antimicrobial peptides.

Emerging evidence shows that the IL-17 pathway is critical in the host defense against various bacterial pathogens [23]; however, very little is known on the exact role of this pathway in* A. baumannii* infection. A recent study examined this in IL-17A KO mice as well as anti-IL-17 neutralization antibody and concluded that IL-17A is not required in primary infection [33]; however, this did not rule out the possibility of a compensatory role of IL-17F, an IL-17 family member that shares the most homology with IL-17A and signals through IL-17RA and IL-17RC complex. Thus, IL-17RA KO mice are required to adequately address this issue. Existing literature also suggested that Th17 cells play a role in vaccine-mediated immunity against* A. baumannii*. The rOmpA vaccine has shown efficacy in animal models through generating antibodies and inducing Th1, Th2, and Th17 responses [34]. However, the roles of each of these specific T helper lineages have not been thoroughly investigated. Furthermore, this study did not measure T follicular helper (Tfh) cell cytokines and markers that are critical in the formation and maintenance of B-cell germinal centers. Nonetheless, immune serum is capable of providing protection upon adoptive transfer, suggesting that a passive immunization strategy can be used in preventing and/or controlling* A. baumannii* infection. Future investigation focusing on the development of monoclonal antibodies against OmpA or other essential proteins from* A. baumannii* should be strongly considered for therapy. Studies using whole cell* A. baumannii* antigen for immunization demonstrated that elevated levels of Th17 polarizing cytokines such as IL-1*β* and IL-6 were observed in nonimmunized mice [35], which correlates with elevated bacterial burden. It could be assumed that these cytokines are also induced by the immunization to promote Th17 responses. The role of such responses should be investigated using KO mice that are deficient in these pathways. A list of commercially available genetic KO mice for studying T helper responses in* A. baumannii* infection is summarized in Table 1. Antimicrobial peptides (AMPs) are another attractive solution in combating* A. baumannii* infection and several AMPs have been shown to have in vitro activities against* A. baumannii* including mastoparan [36] and LL37 [37]. Th17 cytokines including IL-17 and IL-22 are inducers of many AMPs such as *β*-defensin-2, lipocalin 2, and the S100 family proteins from barrier tissues such as gut, skin, and lung [38]. The activities of these AMPs on* A. baumannii* have not been carefully examined. However, Th17 cytokine inducible AMPs could potentially be used in treating antibiotic resistant infections. Indeed, Reg3*γ*, regulated by IL-22, has been shown to be highly effective in killing Methicillin-resistant* Staphylococcus aureus* [39]. The potential role of the IL-17 pathway in* A. baumannii* infections is summarized in Figure 1.

## 5. Conclusions and Perspectives 

The increasing public threat posed by* A. baumannii* infections has greatly intensified clinical and research interest. Significant advances have been made towards understanding the mechanisms of its resistance to antibiotics and hospital hygiene procedures. Accumulating evidence on host-bacterial interactions and the host immune responses could impact available disease treatment. Th17 cells are a critical T-cell subset in controlling Gram-negative bacteria at mucosal barriers and could play a significant role in* A. baumannii* infection. Identifying exact host immune pathways induced by* A. baumannii* infection or immunization will facilitate the discovery of new pharmacological and immunological drug targets to help combat this emerging public health crisis.

## Figures and Tables

**Table 1:** Genetic KO mice that are commercially available for studying *A. baumannii* infection.

Gene products	Th1 pathway	Th2 pathway	Th17 pathway	Tfh pathway
Signature cytokines	Ifng^−/−^ defective in IFN-*γ* production	Il4^−/−^ defective in IL-4 production	IL-17-GFP tracking IL-17A producing cells	
	Il12a^−/−^ defective in Th1 differentiation	Il5^−/−^ defective in IL-5 production	Il17aCre fate mapping IL-17A producing cells	
	Il12b^−/−^ defective in Th1 and Th17 differentiation	Il13^−/−^ defective in IL-13 production	Il22Cre fate mapping IL-22 producing cells	
			Il12b^−/−^ defective in Th1 and Th17 differentiation	

Cytokine/chemokine receptors	Ifngr^−/−^ defective in IFN-*γ* signaling	Il4ra^−/−^ defective in IL-4 and IL-13 signaling		Cxcr5^−/−^ defective in Tfh differentiation

Transcription factors	Tbx21^−/−^ defective in Th1 differentiation	Stat6^−/−^ defective in Th differentiation	Rorc^−/−^ defective in Th17 differentiation	
	Stat4^−/−^ defective in Th1 differentiation			
